# Changes in Tannin Composition of Syrah Grape Skins and Seeds during Fruit Ripening under Contrasting Water Conditions

**DOI:** 10.3390/molecules22091453

**Published:** 2017-09-01

**Authors:** Maria Kyraleou, Stamatina Kallithraka, Nikolaos Theodorou, Pierre-Louis Teissedre, Yorgos Kotseridis, Stefanos Koundouras

**Affiliations:** 1 Laboratory of Enology, Department of Food Science and Technology, Agricultural University of Athens, 75 Iera Odos, 11855 Athens, Greece; mkyraleou@yahoo.gr (M.K.); stamatina@aua.gr (S.K.); ykotseridis@aua.gr (Y.K.); 2 Laboratory of Viticulture, School of Agriculture, Aristotle University of Thessaloniki, 54124 Thessaloniki, Greece; niktheodio@yahoo.gr; 3 Institut des Sciences de la Vigne et du Vin (ISVV), EA 4577, Œnologie, 210 Chemin de Leysotte, University Bordeaux, 33140 Villenave d’Ornon, France; p.teissedre@u-bordeaux2.fr; 4 INRA, ISVV, USC 1366 Œnologie, 210 Chemin de Leysotte, 33140 Villenave d’Ornon, France

**Keywords:** irrigation, proanthocyanidins, flavan-3-ols, galloylation, polymerization, prodelphinidins, terminal and extension subunits

## Abstract

Tannin accumulation and composition were determined in skins and seeds isolated from *Vitis vinifera* cv. Syrah grapes submitted to contrasting water regimes under semiarid climatic conditions. Three irrigation treatments were conducted, starting at berry set through harvest of two growing seasons, 2011 and 2012: irrigation at 100% of crop evapotranspiration ETc (FI), irrigation at 50% of ETc (DI) and non-irrigated (NI). Seed total tannins did not vary with maturity but those of skins underwent a progressive decline (especially in 2011), expressed both on a fresh weight and on a per berry basis. Skin total tannin concentration and content per berry were increased under NI and DI conditions, mainly in 2012. In contrast, seed total tannins (in 2012) and flavan-3-ol monomers and tannin oligomers (both years) were higher in the fully irrigated vines (FI). Skin polymer size increased during ripening, NI and DI skins showing higher mean degree of polymerization (mDP) compared to FI at harvest. NI was also associated with a lower percentage of galloylation (%G) in skin oligomeric fraction (in 2012) and a lower percentage of prodelphinidins in the skin polymeric fraction (both years) at harvest. The mDP and %G of seed extracts did not vary during ripening and were higher in NI but only in 2012. According to the results, management of vine water status was shown to influence tannin amount and composition of Syrah grapes grown under semiarid conditions.

## 1. Introduction

Among grape phenolic compounds, proanthocyanidins or condensed tannins are responsible for the astringent and bitter properties of the wines and are released from both grape skins and seeds [1,2]. Tannins found in grapesare polymers composed of terminal and extension subunits of flavan-3-ols, mainly(+)-catechin (C), (−)-epicatechin (EC), (−)-epicatechin-3-*O*-gallate(ECG) and (−)-epigallocatechin (EGC) linked by C(4)-C(6) or C(4)-C(8) interflavanoid bonds [3]. Grape tannins derived from skins and seeds vary in their relative amount, length, subunit composition and sensory properties. Tannin content of the skins is reported to be lower than seeds [1,4]. Seed tannins are also shorter, with a lower mean degree of polymerization (mDP), while skin tannins are generally larger with a higher mDP [1,5]. Seed tannins are usually composed of C, EC and ECG [6] whereas skin tannins also contain EGC as extension subunit and have a lower proportion of ECG [7]. Epicatechin (EC) is the major extension subunit in the skins, while seeds were found to contain similar amounts of C and EC subunits [8].

The intensity of astringency in wine is reported to be related to both berry tannin concentration [9,10] and composition [11,12]. However, the respective contributions of tannin concentration and composition to wine astringency are not clear. Tannin composition exerted a stronger influence on wine astringency than the total amount of phenolic compounds in some studies [13,14], while others demonstrated that astringency was more correlated with grape total phenolic and tannin content than with tannin structural composition [15]. Astringency was also shown to be dependent on the presence of galloyl groups (%G) and prodelphinidins (proanthocyanidins containing gallocatechin or epigallocatechin subunits). %G values correlated positively with perceived astringency in several studies [4,13] while others either report absence of [12,15] or a negative correlation [16]. Concerning prodelphinidins, their presence is reported to be negatively correlated with astringency perception [14,15,16]. Bitterness is more associated with lower molecular weight compounds such as flavan-3-ol monomers and tannin oligomers [17].

Despite the compositional differences between seed and skin tissues, tannins of both origins were found to be equally astringent when tasted at the same concentration in a wine or buffer medium [18]. However, skin tannins commonly have a higher contribution to the polymer composition of the wine due to the increasing extractability of skin cell walls with the progress of ripening [19] compared to the lignified seed coat [20]. Moreover, the bitter and astringent perception of tannins is also affected by their interactions with soluble polysaccharides present in the grape must [7].

Biosynthesis of tannins occurs after anthesis, reaching a maximum at veraison [21]. Downey et al. [8] observed the highest concentration of flavan-3-ols in Shiraz seeds one week post-veraison, with a subsequent decline until maturity, while in skins, the highest concentration was observed before veraison followed by a continuous decrease until complete ripeness. In other studies, skin tannins of three winegrape varieties were found to change little from veraison to harvest [22] but mDP in Shiraz skin was reported to increase with ripening [23].

The accumulation of phenolic compounds in grapes may be influenced by grape variety [24], environmental conditions [25] and viticultural practices [26]. More notably, the influence of irrigation on the accumulation of anthocyanins in grapes (glycosylated pigments responsible for the color of red wine) has been studied by several authors reporting an overall positive impact of mild water deficit, attributed to changes in berry skin-to-pulp ratio [27] or modifications in grape microclimate [28]. In a previous paper, Kyraleouet al. [29] reported significant increases in Syrah berry skin anthocyanins with water limitation in Greece, but observed that differences were maximum 2–3 weeks after veraison and decreased thereafter to reach similar levels at harvest. However, concerning berry tannins, reports on the effects of water availability are fewer and inconsistent. According to previous studies, water deficit is reported to have little direct effect on the accumulation of tannins in berries [30,31]. In other studies, water limitation decreased the amount of seed flavan-3-ols at harvest in Cabernet Sauvignon [32], while, according to Chacón et al. [33], the concentration of flavan-3-ols and tannins in Merlot seeds increased with the magnitude of water deficiency. Genebra et al. [34] found no impact of irrigation on the levels of Tempranillo seed tannins, although several genes of the biosynthetic pathway of flavan-3-ols were up-regulated, while Zarrouk et al. [35] found an increasing trend of skin tannins with irrigation.

Greece is generally characterized by warm to hot climate conditions for winegrape production and recent studies have reported increasing trends in summer temperatures leading to more frequent and intense drought events [36] thereby increasing the dependence of viticulture on irrigation. Moreover, there is limited information available concerning the effect of irrigation on the compositional characteristics of grape tannins. According to Ollé et al. [21], mDP values of Shiraz berry tannins remained unaffected by water regime and similar results were reported for Cabernet Sauvignon by Kennedy et al. [30]. Kyraleou et al. [15] investigated the effect of irrigation on the astringency perception of Syrah grape seed and skin extracts in a model solution, by sensory analysis. According to their results, seed and skin extracts of fully irrigated vines were perceived significantly more astringent than those of non-irrigated ones (with the exception of skin extracts of 2012) suggesting that the manipulation of water regime might be a valuable tool for the optimization of red wine sensory properties. The aim of the present work was to extend our previous investigations and to study the effect of irrigation on the compositional properties of tannins located in skins and seeds of Syrah grapes over the whole ripening period, under the typical semiarid climatic conditions of Northern Greece.

## 2. Results and Discussion

### 2.1. Vine Water Status and Growth

Results of irrigation effects on vine water status and growth were reported in detail in a previous work [29] and are summarized in the Table S1. Regarding the two years, the drier 2012 was characterized by a more limiting water status than 2011, leading to higher berry temperatures at midday; 2011 was characterized by overall higher yields and berry size. Predawn water potential (Ψ_d_) was affected by irrigation regime and differences were consistent among irrigation treatments in both years, with more negative values for the non-irrigated vines (NI), intermediate for the deficit-irrigated ones (DI) and higher values for fully irrigated vines (FI). Limited water availability under NI conditions increased midday berry temperature (T_b_), possibly due to increased direct exposure of grapes to incident light related to greater canopy porosity [28]. The lower vigor of non-irrigated vines was manifested by the lower winter pruning weights in both years, compared to irrigated treatments. NI vines had lower berry weight at harvest, but no difference was detected for yield per vine. Finally, sugar levels in the grape must were responsive to water conditions only in 2011 when NI plants showed the higher total soluble solids at harvest.

### 2.2. Total Tannins of Skins and Seeds

**Figure 1:**
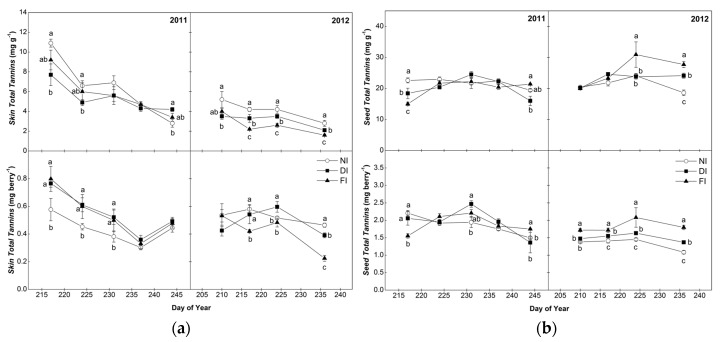
Seasonal pattern of total tannin (TT) concentration (mg catechin g^−1^ fresh weight) and content (mg^−1^ catechin berry) of skins (**a**) and seeds (**b**) of Syrah grapes in the three irrigation treatments (FI, 100% of ETc; DI, 50% of ETc and NI, non-irrigated) in 2011 and 2012. Bars indicate ±S.E. of the mean value. Values with different letters within samplings are significantly different (Tukey’s test, *p* < 0.05). Harvest data for TT concentration were previously reported in Kyraleou et al. [15].

Total tannin (TT) concentration and content per berry of the skin and seed extracts for the years 2011 and 2012 are presented in Figure 1. In general, TT concentration and content per berry of seeds was higher than the skins in agreement with other studies [31,37,38]. Comparing vintages, skin samples harvested in 2011 were richer in TT than 2012, while seed samples presented similar TT between years (Figure 1). Considering tannin seasonal pattern, a decreasing trend during berry ripening was highlighted for skin TT concentration and content in both years (*p* < 0.001 for 2011 and *p* < 0.01 for 2012), similarly to previous studies [5,37]. On the contrary, seed TT changed very little from veraison to harvest in contrast to previous reports of a general decline of seed tannins during ripening associated with seed coat browning [23,24,37]. Irrigation affected both the concentration (amount per unit skin and seed fresh weight) and the absolute content (amount per berry) of skin and seed phenolics. In 2011, TT concentration of NI skin tissues was significantly higher compared to those from the irrigated treatments until prior to harvest (when it was not different from FI); however, when the results were expressed on a per berry basis, NI berries had the lowest TT content (amount per berry) in the beginning of the season but differences were absent at harvest (Figure 1). Water deficit may increase phenolic concentration due to its effect on berry size [38] by selectively increasing the absolute mass of skin tissue [39] rather than a direct biosynthetic effect. In 2012, both TT concentration and amount per berry were higher under NI and DI conditions, showing a positive biosynthetic effect of water deficit on skin berry tannin content. A significant negative correlation between predawn water potential (Ψ_d_) measurements (E-L stages 33, 35, 36 and 38) and skin TT at harvest was found in 2012 (Supplemental Table S2) while a similar but insignificant trend was also observed in 2011. For E-L 33 and E-L 36 in 2012, TT concentration was positively correlated at the same time with berry temperature recorded at midday (Table S3). T_b_ was consistently higher in NI in both years, most probably as a result of the higher light penetration in the cluster zone due to the reduced canopy density as shown by the lower values of pruning weight (Supplemental Table S1) [28].

For seed TT, an irregular pattern in relation to water status was observed in 2011, with no consistent differences among irrigation treatments (with the exception of harvest sampling when FI demonstrated superior values); however, in 2012, both the concentration per seed fresh weight (end of ripening period) and the content per berry (whole ripening period) of total tannins were higher in FI seed samples than NI, with DI presenting intermediate values (Figure 1). This observation was further strengthened by a highly significant positive correlation (Pearson’s correlation coefficient 0.90) between Ψ_d_ at veraison and harvest and seed TT at harvest in 2012 (Supplemental Table S2). In previous studies, water regime did not alter seed tannin content in Shiraz [31] while Casassa et al. [38] reported increased levels of seed tannins under both early season and continuous water deficit in Cabernet Sauvignon grapes. The decreased seed TT concentration and content under NI and DI conditions during the late part of 2012 season might also be related to the increased temperature of NI and DI berries (Supplemental Table S1); according to Bonada et al. [31], heating of grapes reduced seed tannins by 20% compared to those under ambient conditions. In the conditions of this experiment, seed TT at harvest was negatively correlated with T_b_ measured at E-L stages 33 (green berry) and 36 (berries half-ripened) during 2012 (Supplemental Table S3).

### 2.3. Flavan-3-ol Monomers and Tannin Oligomers of Skins and Seeds

**Figure 2:**
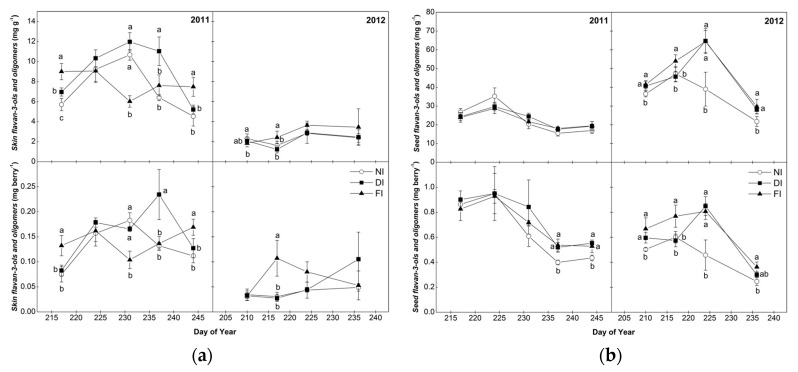
Seasonal pattern of total flavan-3-ol monomers and tannin oligomers concentration (mg catechin g^−1^ fresh weight) and content (mg^−1^ catechin berry) of skins (**a**) and seeds (**b**) of Syrah grapes in the three irrigation treatments (FI, 100% of ETc; DI, 50% of ETc and NI, non-irrigated) in 2011 and 2012. Bars indicate ±S.E. of the mean value. Values with different letters within samplings are significantly different (Tukey’s test, *p* < 0.05). Harvest data were previously reported in Kyraleou et al. [15].

Flavan-3-ol monomers [(+)-catechin (C), (−)-epicatechin (EC), epicatechin-3-*O*-gallate (ECG), (−)-epigallocatechin (EGC)] and tannin oligomers (procyanidins B1, B2, A2 and C1) were more abundant in berry skins in the organic fractions from 2011 samples than 2012 (Figure 2). However, no consistent pattern could be evidenced in any year for the seasonal evolution of skin flavan-3-ol monomers and tannin oligomers concentration and content, contrary to skin TT (Figure 1). The different pattern during ripening between skin flavan-3-ol monomers and tannin oligomers and TT may be due to the fact that skin tannins mostly consist of polymers, monomers/oligomers representing a small portion of skin TT.

No clear outcome was observed on the effect of irrigation on the levels of monomers/oligomers in the skins, in either year (Figure 2), although NI grapes in 2011 displayed a tendency toward lower values than FI, with a significant positive correlation observed between the concentration of trimer C1 and Ψ_d_ (Supplemental Table S2) in 2011. Changes in water conditions can result in modifications in cluster microclimate (i.e., a decrease in water potential can lead to increased berry temperature [28] due to more open canopy in the fruit zone). Accordingly, significant negative trends of EGC, B2, C1 and A2 at harvest with midday T_b_ were observed in 2011 (Supplemental Table S3) suggesting a decreasing amount of some skin flavan-3-ol monomers and tannin oligomers with the rise in berry temperature, related to water limitation. Increased berry temperatures may be detrimental to flavonoid synthesis, especially under semiarid conditions as reported previously [40].

Regarding seed samples, the concentration of flavan-3-ol monomers and tannin oligomers was higher than in skins in both years (Figure 2). In both years, the concentration of seed flavan-3-ol monomers and tannin oligomers increased initially and then declined until harvest [32,41]. Maximum accumulation was attained earlier in 2011 than in 2012 (except for NI). However, sampling date effect was significant at *p* < 0.001 across treatments, in 2011 but not in 2012 (data not shown). Downey et al. [8] also reported maximum flavan-3-olmonomers accumulation in Syrah seeds one week post-veraison followed by a sharp decrease during the succeeding three weeks. Previous works have suggested that the decline of tannins in the seeds after veraison could be related to oxidative cross linking of polymers [42]. Since monomers and dimers are responsible for the bitter sensation compared to polymers [17], the reduction in seed flavan-3-ol monomers and tannin oligomers with grape ripening suggests that the ripening process is expected to be associated with a decline of bitterness in the final wine.

Irrigation in 2011 did not affect flavan-3-ol monomers and tannin oligomers content of the seed samples at harvest; however, monomers/oligomers amount per berry was lower in NI than in the irrigated vines (Figure 2). In 2012, FI and DI seeds contained significantly higher levels of total monomers/oligomers (expressed both as concentration per seed fresh weight and as content per berry) compared to NI, mainly at maximum accumulation point but differences remained significant until harvest (Figure 2), which is in agreement with our results on seed TT (Figure 1). Regarding the trends of individual flavan-3-ol monomers with irrigation, a significant positive correlation with Ψ_d_ was observed for seed C concentration, in 2012 (Supplemental Table S2). According to other studies [7,32], seed flavan-3-ol monomers of Cabernet Sauvignon and Merlot were drastically reduced under minimal water availability. Differences among irrigation treatments in seed flavan-3-ols may be explained by vigour-related changes in canopy microclimate. Grapes in FI were probably less exposed to direct sunlight due to greater canopy size, as could be assumed by their lower midday temperature compared to the NI grapes (Supplemental Table S1). These findingsare in agreement with previous studies reporting a higher amount of seed flavan-3-ol monomers in shaded grapes [43]. However, in the conditions of this study, T_b_ was not related to the changes in seed phenolic parameters in either year (Supplemental Table S3).

### 2.4. Composition of Skin and Seed Tannins

#### 2.4.1. Percentage of Galloylation (%G)

Regarding %G values (Table 1), seeds were characterized by higher average values than skins in both oligomers and polymers, in agreement with the findings of other researchers for the varieties Cabernet Sauvignon, Merlot [1], Plavac mali, Babić [4] and Aglianico [44]. The oligomeric fractions of both skins and seeds showed statistically higher values of %G than the respective values of polymers. The effect of year was evident only for the seed extracts in both fractions studied, with 2011 samples having higher %G values than the respective 2012 samples (*p* < 0.001, across samplings and treatments). During ripening phase, %G was quite constant (Table 1). A decreasing trend of %G was only observed in seed oligomeric fraction, mostly during ripening of 2011. Bordiga et al. [5] observed a decreasing trend in %G values of the seed fractions in three of the six varieties they studied while Obreque-Slier et al. [33] observed a decrease of %G at ripeness which was however followed by an increase during over-maturity. %G was significantly lower in NI skin oligomeric fraction at harvest in both years, compared to DI and FI, while the opposite occurred for seed oligomers (Table 1) where NI was associated with the higher %G at harvest of 2012.

#### 2.4.2. Mean Degree of Polymerization (mDP)

**Figure 3:**
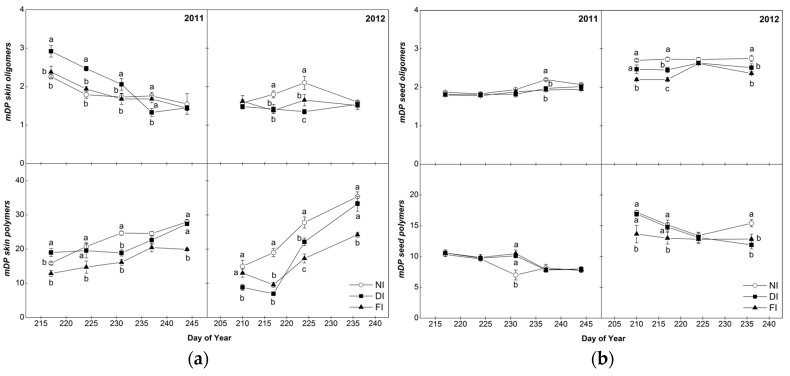
Seasonal pattern of mDP of oligomeric and polymeric fractions of Syrah grape skins (**a**) and seeds (**b**) in the three irrigation treatments (FI, 100% of ETc; DI, 50% of ETc and NI, non-irrigated) in 2011 and 2012. Bars indicate ±S.E. of the mean value. Values with different letters within samplings are significantly different (Tukey’s test, *p* < 0.05). Harvest data were previously reported in Kyraleou et al. [15].

The average of mDP of the polymeric fraction of skins were two-times higher than the corresponding values of seeds, in agreement with previous studies related to six grape varieties [5] whereas the mDP range of the oligomeric fraction was similar between seeds and skins (Figure 3). In general, in the skins, oligomers and polymers represented 2–7% and 93–97% of the extracted tannins respectively, whereas in the seeds, oligomers represented 22–29% and polymers 71–78%, depending on the sampling day (data not shown).

In the skin samples, mDP was similar, on average, between years (Figure 3). The mDP of skin oligomeric fraction showed a slight decreasing trend during ripening in 2011 (*p* < 0.001 for sampling effect across treatments) while, in 2012, despite some fluctuations, mDP values remained approximately constant (*p* = 0.375 for sampling effect across treatments). In contrast to skin oligomers, the mDP of the polymeric fraction of skin tannins increased in both years (*p* < 0.001 for sampling effect in both years, across treatments) during the ripening season (Figure 3) confirming previous studies [5,30,37]. Between veraison and harvest, mDP increased, on average across treatments by 57% in 2011, and it showed a 3-fold increase in 2012. As a result, the polymeric tannins were increased during maturation in skins, while oligomers were decreased (data not shown). Since polymeric compounds are predominant in the skins, ripening process would be expected to be associated with an increase in astringency perception [17]. Regarding the effect of irrigation on the polymerization of Syrah skin tannins, NI and DI were characterized by higher mDP in the polymeric skin fractions while fewer differences among treatments were recorded for the oligomeric fraction, especially at harvest (Figure 3). According to Chira et al. [1], polymeric compounds are increasingly reactive with proteins with increasing mDP, therefore our results suggest a higher astringency potential of Syrah skins under NI conditions.

Seed tannin mDP values were, on average, higher in 2012 than 2011, for both oligomeric and polymeric fractions (Figure 3) and remained approximately constant throughout ripening in both years [41]. Contrary to our results, previous works observed a decreasing trend in seed mDP values during berry ripening phase in different grape varieties [5,32]. The mDP values of the seed tannins did not show any clear dependence on irrigation in 2011 whilst in 2012, NI seeds had a higher mDP than FI and DI ones for both the oligomeric and polymeric fractions (Figure 3).

Regression analysis showed a highly significant power correlation between %G and mDP (Supplemental Figure S1), for both skins (*y* = 10.96*x*^−1.27^, r = 0.948, *p* < 0.001) and seeds (y = 17.51x^−0.69^, r = 0.921, *p* < 0.001). Specifically, for skin tannins, %G higher than 2.5 was associated with tannin monomers and oligomers (mDP < 4). Inversely, mDP higher than 8 was associated with an absence of ECG subunits in skin tannins. A similar trend was observed in seeds, with high %G (superior to 6) associated only with monomers, dimers and trimers while larger molecules (mDP > 6) presented a lower percentage of galloylation (%G < 5). According to these results, larger tannins of both skins and seeds are associated with a low percentage of ECG subunits.

#### 2.4.3. Percentage of Prodelphinidins (%P)

Another feature of tannin composition which is associated with astringency perception is the molar percentage of prodelphinidins (proanthocyanidins containing flavanols with trihydroxylation on the B-ring, namely gallocatechin or epigallocatechin subunits, %P) [14,15,16].

**Figure 4:**
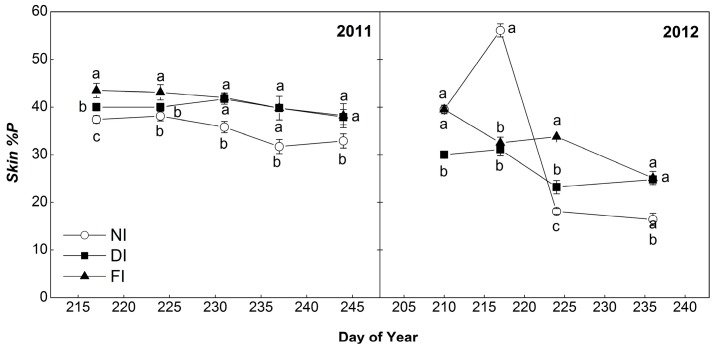
Seasonal pattern of %P of Syrah grape skin tannins in the three irrigation treatments (FI, 100% of ETc; DI, 50% of ETc and NI, non-irrigated) in 2011 and 2012. Bars indicate ±S.E. of the mean value. Values with different letters within samplings are significantly different (Tukey’s test, *p* < 0.05).

According to our results (Figure 4), NI berries presented a lower %P in skin polymer fraction during the second half of ripening period, with significant differences at harvest with the irrigated treatments (32.9% compared to 38.2% and 37.9% for FI and DI respectively in 2011, and 16.4% compared to 25.1% and 24.8% for FI and DI respectively in 2012). Regression analysis showed a significant quadratic correlation between %P and mDP (Supplemental Figure S2), for skin polymeric fraction (*y* = −0.062*x*^2^ + 2.061*x* + 21.38, r = 0.728, *p* < 0.001) according to which, for mDP higher than 10, %P followed a decreasing pattern with increasing mDP, i.e., larger molecules had a lower percentage of prodelphinidins.

#### 2.4.4. Subunit Composition

Table 2 and Table 3 show the evolution during ripening of the composition of terminal and extension subunits in polymeric and oligomeric fractions of skin and seed tannins obtained after phloroglucinolysis (C, EC, ECG and EGC). In the skin extracts, EC was the main terminal and extension subunit, accounting in oligomers for over 80% of total subunits at harvest during both growing seasons, while in polymers a substantial amount of EGC (30–40% of total) was also recorded but only as extension subunit (Table 2). Moreover, EC ratio in the total extension subunits of skin polymers tended to increase with the progress of ripening while that of EGC followed an opposite pattern (Table 2). Catechin (C) proportion in skins was <10% of total subunits, with the exception of a higher proportion in terminal subunits of both oligomers and polymers, in 2011. Epicatechin-3-*O*-gallate (ECG) had the smallest contribution and was almost entirely absent from skin polymeric fraction (Table 2).

Our findings concerning the subunit composition of skin polymers and oligomers are consistent with earlier work that reported EC as the predominant subunit of skin tannins [5]. However, Obreque-Slier et al. [37] did not determine EC in terminal subunits of Carménère and Cabernet Sauvignon skins while other authors found C as the main terminal and/or extension subunit in grape skins in a number of varieties [6,26]. Epigallocatechin (EGC) as a terminal subunit was detected in a few studies [44] or it was entirely absent [1,6], in agreement with the results of this study.

In the seed oligomeric fraction, EC made up, on average, 53.3% and 62.15% of terminal subunits at harvest, and 42.9% and 60.8% in the polymeric fraction ,in 2011 and 2012 respectively (Table 3). Catechin (C) had the second highest proportion, representing at harvest (on average across years) 25.9% and 28.6% of the total terminal subunits of oligomers and polymers respectively, followed in order by ECG. By contrast, in extension subunits, EC was the dominant subunit (>88% of the total) in both oligomers and polymers, while C accounted for less than 10% and ECG contributed a minor percentage (Table 3). Epigallocatechin (EGC) was not detected in the oligomeric and polymeric fraction of the seeds.

Epicatechin (EC) was found as the dominant terminal subunit in Carménère seed tannins [37], but in other varieties [4,5,41], C was the main unit in seed terminal subunit composition. However, previous studies involving different varieties confirm our results that EC is the main extension subunit in oligomeric and polymeric seed fractions [4,5,37]. A decreasing trend with ripening for ECG and an increasing one for EC were observed in previous cases for seed terminal subunits [42]. In this study, C, EC and ECG relative proportions remained constant during the season (Table 3). Moreover, in 2012, EC percentages were higher compared to those in 2011 in all cases. Bordiga et al. [5] also observed a general decrease of C and ECG percentages and an increase of EC as terminal subunits in most of the six varieties they analyzed.

Regarding irrigation effects on tannin subunit composition, NI skins polymeric fraction showed a higher proportion of EC and a lower proportion of EGC at harvest in extension subunits, as compared to DI and FI (Table 2). The lower ratio of EGC to EC in NI Syrah grape skin polymeric fraction might imply an increase in the perception of astringency in the wines produced from water-stressed vines based on the results of some studies [15,16]. In seed tannins, a significant effect of irrigation was observed at harvest mostly in 2011, with higher C percentage in terminal and extension subunits in both fractions for NI; in contrast, EC percentage in terminal subunits was higher in both fractions in FI (Table 3).

Comprehensive investigations on the relation between grape tannin content and composition with wine or grape sensory properties are rather fragmentary and the results are often contradictory. Several published articles have examined either the influence of total tannin or phenolic content [9,10] or the effect of tannin composition [12,45,46] on grape or wine astringency. However, even when both tannin concentration and composition have been examined simultaneously, the results are frequently conflicting. Chira et al. [13] reported that mDP exhibited a higher influence on wine astringency compared with total phenolic compounds or total tannins. In contrast, the results presented by Quijada-Morin et al. [14] showed that astringency in wine is more affected by subunit composition than by the total concentration whereas according to other studies [15], astringency was more correlated with grape total phenolic and tannin content than tannin structural composition. However, recently, purified and certified oligomeric tannins compounds were observed in a salivary protein binding test by HPLC-FLD and a positive relationship between degrees of polymerization (DP) (ranged from one to five) and salivary protein binding affinity was discovered [47]. Controversies have been also reported in the literature regarding the role of galloyl groups (%G) in the astringency perception. %G values correlated positively with perceived astringency in several studies [4,13] while others either report absence of [12,15] or negative correlation [16]. In the case of skin EGC, most of the published data are in agreement that it is negatively correlated with astringency perception [11,14,15]. A significant influence of extraction method and grape variety was also stated [44], principally as regard the percentage of prodelphinidins (%P) for skins, and of galloylation (%G) for seeds.

According to the results, total tannins were higher in Syrah seeds compared to skins; moreover, seed TT were variable while those of skins showed a decreasing pattern during ripening. The decline in TT in skin tissues during ripening was probably not related to any dilution effect driven by the increase in berry size, since it was also observed for TT amount per berry (Table 1). This decrease was probably caused, as already reported for Syrah [8], by stable associations between tannins and other cellular components such as cell wall polysaccharides, lignins and proteins. Seed tannins were also shorter, with a higher degree of galloylation and with subunit composition consisting mainly of EC as extension and terminal subunit [37]. In the skins, tannins were larger, increasing mDP with ripening, and less galloylated, consisting mostly of EC terminal subunits and EC and EGC extension subunits [5,26,37]. In a previous work published from the same experiment [15], Syrah skin extracts were perceived less astringent than those of seeds, despite their higher mDP. The lower contribution of skin tannins to the total tannin content of berries in the conditions of our experiment (weighing 0.2 to 0.8 mg per berry, compared to 1.2 to 2.5 mg of seed tannins), might provide an explanation for this result. Moreover, according to the findings of Quijada-Morin, et al. [48], the extraction of tannins becomes more difficult as their degree of polymerization increases, thus, larger skin tannins may be less easily released from skins, thereby limiting their contribution to the astringent perception of the wine.

As regards the irrigation effect on tannin concentration and content, NI conditions increased skin tannin concentration (both years), but this was independent of any berry-size related effect only in 2012. Concerning irrigation effects on tannin composition of skin samples, NI conditions increased tannin polymerization and decreased the percentage of prodelphinidins in the polymeric fraction (both years); we could therefore speculate that grape skins of non-irrigated vines would be expected to confer a higher astringency sensation to the wines compared to those of FI ones, based on their compositional characteristics [11,17]. However, in a previous paper from the same experiment [15], skin extracts in a model solution from NI vines were perceived significantly less astringent by sensory analysis than those from FI skins in 2011, while no effect of irrigation was observed in 2012. In the case of seeds, non-irrigated conditions tended to increase %G and mDP of tannins (only in 2012) but it was found to reduce the content per berry of seed tannins and flavan-3-ol monomers and tannin oligomers (especially in 2012), which might explain the lower astringency of seed extracts of NI vines as perceived in the sensory analysis in a previous paper from the same experiment [15].

## 3. Materials and Methods

### 3.1. Chemicals

Ethyl acetate, chloroform, methanol, ethanol, acetone were of HPLC grade, sodium metabisulfite, sodium carbonate, phloroglucinol, l-(+)-tartaric acid, (+)-catechin, l-ascorbic acid, hydrochloric acid (37%), sodium hydroxide, acetic acid, were purchased from Sigma Aldrich (Saint Louis, MO, USA). Bovine serum albumin (Albumin Fraction V) was from Applichem (Darmstadt, Germany). (−)-Epicatechin, (−)-epigallocatechin, (−)-epicatechin-3-*O*-gallate, and proanthocyanidins B1, B2, C1 and A2 were purchased from Extrasynthese (Genay Cedex, France).

### 3.2. Experimental Conditions

The study was carried out during two growing seasons (2011 and 2012) in a commercial vineyard located in Epanomi, Northern Greece (40°45′ N, 22°92′ E, 150 m altitude), planted with *Vitis vinifera* L. cv. Syrah onto 1103P rootstock. Three irrigation treatments were replicated three times, starting at berry set through harvest: full irrigation (FI), receiving 100% of crop evapotranspiration (ETc), deficit irrigation (DI) receiving 50% of ETc and non-irrigated (NI). 2012 was characterized by warmer weather than 2011 (average temperature for the April to September period was 22.0 °C in 2012 compared to 20.5 °C in 2011). On the contrary, 2012 was dryer, with a total rainfall for the growing season of 93 mm, compared to 159 mm in 2011. Experimental conditions were presented in detail in Kyraleou et al. [29].

### 3.3. Extraction of Phenolic Compounds from Grape Seeds and Skins

In 2011, five berry samplings took place at Day of Year (DOY) 217, 224, 231, 237 and 244 and, in 2012, four samplings at DOY 210, 217, 224 and 236, starting after veraison was completed (50% veraison occurred at DOY 207 in 2011 and DOY 206 in 2012). The skins and seeds of 150 berries were manually removed. Then they were freeze-dried and finally were grounded to obtain powder. Tannin extraction was performed in triplicate according to a previously published method [1]. A 3 g portion of the obtained powder was firstly extracted with 25 mL mixture of acetone/water (80:20, *v*/*v*) for 3 h and then with 25 mL mixture of methanol/water (60:40 *v*/*v*) for 2.5 h. The centrifugal supernatants were combined and evaporated under reduced pressure at 30 °C to remove organic solvents; the residue was dissolved in water and lyophilized to obtain a crude tannin extract. The extracts were then subjected to freeze-drying and they were grounded to obtain fine powder.

### 3.4. Grape Tannin Content

Part of the obtained fine powder was dissolved in a model solution (12% ethanol; 5 g/L tartaric acid; pH 3.5 adjusted with 1 N NaOH) at a concentration of 10 g/L. For the total tannin estimation, the protein precipitation assay using bovine serum albumin-BSA [49] was employed. A 500 μL portion of the sample was reacted with 1 mL of BSA. After incubation for 15 min, the sample was centrifuged for 10 min at 12,000 rpm to pellet the protein-tannin precipitate. The protein-tannin pellet from tube was washed with 250 µL of the acetic acid/NaCl buffer (pH 4.9) and the sample was centrifuged for 5 min at 12,000 rpm. The wash solution was discarded, then 875 µL of a buffer containing 5% TEA (*v*/*v*) and 10% SDS (*w*/*v*) was added and then the absorbance at 510 nm was determined (OD1). Then, 125 µL of ferric chloride reagent (10 mM FeCl_3_ in10 mM HCl) was added and the absorbance determined after a 10-min incubation (OD2). Absorbance measurements were recorded on a V-530 UV/VIS spectrophotometer (Jasco, Victoria, BC, Canada). The tannin concentration was calculated as the absorbance OD2 minus the OD1 and expressed in catechin equivalents by comparison with a standard curve prepared with catechin. All analyses were performed in triplicate (for every sample).

### 3.5. TanninAnalysis

Tannins were analyzed according to the methods described in previous studies [1,24,26]. A part of the remaining crude extract of seeds and skins was dissolved in a 5% of aqueous ethanol solution (10 mL) at a concentration of 80 g/L and extracted three times with chloroform to remove the lipophilic material. The chloroform was discarded after centrifugation and the aqueous phase was extracted three times with ethyl acetate (3 × 10 mL). Organic and aqueous fractions were collected separately; the organic fraction was concentrated under reduced pressure at 30 °C and both fractions lyophilized to obtain dry powder. Briefly, the following monomeric and oligomeric phenolic compounds [(+)-catechin (C), (−)-epicatechin (EC), epicatechin-3-*O*-gallate(ECG), (−)-epigallo-catechin (EGC), and procyanidins B1, B2, A2 and C1] were determined by HPLC [24]. The tannin mDP, %P and %G were quantified by phloroglucinolysis [25,50]. Tannin extracts were re-dissolved in methanol (5 g/L) and were left to react with phloroglucinol solution (50 g/L phloroglucinol, 10 g/L ascorbic acid, 0.1N HCl, in methanol) for 20 min at 50 °C. The reaction was terminated by addition of 1 mL aqueous sodium acetate (40 mM). The response factors used for the calculations of tannin composition characteristics were taken from Drinkine [51]. In more detail, the following response factors were used (L/mmol): 19,433, 60,585, 60,585 and 19,433 for EGC, C, EC and ECG phloroglucinol adducts respectively while 19,052, 57,156, 57,156 and 197,204 were employed for EGC, C, EC and ECG respectively. The mDP, %P and %G were calculated using the following equations:

mDP = (Σ terminal subunits + Σ extension subunits)/Σ terminal subunits %G = [(ECGe + ECGt)/(Σ terminal subunits + Σ extension subunits)] × 100 %P = [(EGCe + EGCt)/(Σ terminal subunits + Σ extension subunits)] × 100

where Σ terminal units: sum of all terminal subunits released by phloroglucinol as flanan-3-ols (expressed as mmol) and Σ extension units: sum of all extension subunits (expressed as mmol).

Reaction products were analyzed on an LC-MS 2010A instrument coupled to a single quadrupole mass spectrometer equipped with an electrospray ion source (Shimadzu, Kyoto, Japan). The mass spectrometer was operated in positive-ion mode. The source’s temperature was set at 70 °C, the capillary voltage at 3.5 kV and the cone voltage at −30 eV. The absorbance was recorded at 280 nm and mass spectra were recorded in the range of 50–1500 amu. Separation was performed on an XTerra RR C18 (100 × 4.6 mm, 3.5 μm) reversed-phase column (Waters, Milford, MA, USA) at a flow rate of 0.5 mL/min, using a 20 μL injection volume and the following elution program: eluent A from 80% to 40% in 20 min, which was kept isocratic for further 10 min and then from 40% to 80% in 2 min [26]. Eluent A was 0.1% aqueous acetic acid and eluent B methanol. All analyses were performed in triplicate (for every sample).

### 3.6. Statistics

Data were subjected to repeated measures ANOVA and one-way ANOVA (between irrigation treatments on a single date), on Statistica V.7 Software (StatsoftInC., Tulsa, OK, USA). Comparison of mean values was performed using Tukey’s HSD test when samples were significantly different after ANOVA (*p* < 0.05). Pearson’s correlation analysis was used to investigate relationships between phenolic composition and predawn water potential (Ψ_d_) and berry temperature (T_b_).

## 4. Conclusions

Management of vine water status was found to alter the composition characteristics of skin and seed tannins; full irrigation induced a lower degree of skin tannin polymerization (both years) while full and deficit irrigation induced a lower degree of tannin polymerization in the seeds (in 2012), a higher percentage of prodelphinidins in skins (both years) and a lower percentage of galloylation in the seeds (in 2012). However, astringency intensity in wine is also determined by tannin concentration and, most importantly, their amount per berry at harvest. In the conditions of this experiment, irrigation increased seed tannins and flavan-3-ol monomers and tannin oligomers concentrations and amount, mainly in 2012; in the skins, tannin content was increased in the non- and deficit irrigated vines, but only in 2012. 

Under the conditions of Greek viticultural areas, and in view of the observed shifts towards warmer and drier future climatic conditions, reducing water usage is a priority. According to the results of this experiment, water deficits during berry development (as in the non-irrigated treatment) seem to produce grapes with a lower seed tannin content at harvest but possibly with higher skin tannins (which are characterized by compositional traits considered to enhance astringency sensation e.g., increased polymerization). However, since seed tannins are more plentiful per berry than those of skins, deficit water conditions would be probably expected to decrease wine astringency.

HHFurther research is needed to better understand the complex effects of various environmental and viticultural conditions on the accumulation and composition characteristics of skin and seed tannins as well as their direct effect on the actual sensory properties of wines, across a wide range of cultivars.

## Figures and Tables

**Table 1:** Seasonal evolution of %G of oligomeric and polymeric fractions of skins and seeds of Syrah grapes from the three irrigation treatments (FI, 100% of ETc; DI, 50% of ETc and NI, non-irrigated) in 2011 and 2012.

		Day of 2011					Day of 2012			
		217	224	231	237	244 ^2^	210	217	224	236 ^2^
**Skins**										
**Oligomers**	**NI**	4.88 ± 0.15 a ^1^	6.74 ± 0.20 a	8.17 ± 0.68 a	6.35 ± 0.30 b	6.74 ± 1.10 b	6.52 ± 0.24 ab	4.98 ± 0.44 a	4.20 ± 1.96 b	3.77 ± 0.58 c
	**DI**	3.55 ± 0.15 b	3.02 ± 0.16 b	8.03 ± 0.62 a	3.73 ± 0.11 c	8.63 ± 0.32 a	4.55 ± 0.31 b	4.25 ± 0.12 a	13.55 ± 1.26 a	5.44 ± 0.55 b
	**FI**	4.23 ± 0.30 ab	6.67 ± 0.32 a	6.05 ± 1.00 b	9.51 ± 0.49 a	8.04 ± 0.50 a	8.38 ± 1.39 a	6.11 ± 1.18 a	9.47 ± 1.72 ab	8.97 ± 3.22 a
**Polymers**	**NI**	0.19 ± 0.01 b	0.29 ± 0.03 a	0.34 ± 0.01 a	0.16 ± 0.03 b	0.25 ± 0.00 a	0.18 ± 0.02 a	0.19 ± 0.03 a	0.36 ± 0.02 a	0.31 ± 0.02 b
	**DI**	0.23 ± 0.01 b	0.21 ± 0.03 a	0.21 ± 0.03 b	0.17 ± 0.00 b	0.23 ± 0.02 a	0.34 ± 0.18 a	0.18 ± 0.03 a	0.31 ± 0.00 a	0.36 ± 0.02 a
	**FI**	0.28 ± 0.02 a	0.22 ± 0.02 a	0.13 ± 0.00 c	0.32 ± 0.03 a	0.26 ± 0.01 a	0.15 ± 0.01 a	0.18 ± 0.03 a	0.42 ± 0.09 a	0.23 ± 0.03 c
**Seeds**										
**Oligomers**	**NI**	16.19 ± 0.34 a	14.93 ± 0.39 a	12.51 ± 0.22 a	11.88 ± 0.26 a	10.25 ± 0.25 a	7.72 ± 0.06 a	7.90 ± 0.12 a	7.12 ± 0.16 a	6.91 ± 0.15 a
	**DI**	15.55 ± 0.28 a	14.26 ± 0.21 a	11.60 ± 0.96 a	11.52 ± 0.15 ab	10.70 ± 0.21 a	7.77 ± 0.19 a	7.79 ± 0.11 a	7.13 ± 0.23 a	6.41 ± 0.03 b
	**FI**	16.06 ± 0.50 a	14.48 ± 0.23 a	13.20 ± 0.10 a	10.64 ± 0.39 b	10.24 ± 0.05 a	6.98 ± 0.27 a	7.06 ± 0.12 b	6.85 ± 0.62 a	6.25 ± 0.08 b
**Polymers**	**NI**	5.07 ± 0.19 a	4.69 ± 0.05 a	4.57 ± 0.43 a	5.26 ± 0.18 a	5.20 ± 0.08 a	2.18 ± 0.02 a	2.13 ± 0.07 a	2.18 ± 0.04 ab	2.36 ± 0.05 a
	**DI**	5.16 ± 0.14 a	4.74 ± 0.14 a	4.16 ± 0.39 a	5.36 ± 0.18 a	5.25 ± 0.16 a	2.28 ± 0.10 a	2.17 ± 0.15 a	2.28 ± 0.03 a	2.36 ± 0.04 a
	**FI**	5.16 ± 0.14 a	4.78 ± 0.17 a	4.84 ± 0.19 a	4.91 ± 0.12 a	5.17 ± 0.08 a	2.70 ± 0.44 a	2.18 ± 0.05 a	2.11 ± 0.03 b	2.31 ± 0.09 a

^1^ Values with different letters within samplings and fractions are significantly different (Tukey’s test, *p* < 0.05). ^2^ Harvest data were previously reported in Kyraleou et al. [15].

**Table 2:** Seasonal evolution of the percentage of terminal and extension subunits in oligomeric and polymeric fractions of Syrah grape skins from the three irrigation treatments (FI, 100% of ETc; DI, 50% of ETc and NI, non-irrigated) for 2011 and 2012. Terminal subunits: Ct, (+)-catechin; ECt, (−)-epicatechin; ECGt, epicatechin-3-*O*-gallate; extension subunits: %Ce, (+)-catechin; %ECe, (−)-epicatechin; %ECGe, epicatechin-3-*O*-gallate; %EGCe, (−)-epigallocatechin;nd, not detected.

		Day of 2011					Day of 2012			
		217	224	231	237	244	210	217	224	236
**Oligomers**										
**Ct**	**NI**	20.7 ± 0.56 b ^1^	11.1 ± 0.38 c	10.9 ± 0.17 c	8.1 ± 0.50 a	6.5 ± 0.52 a	10.3 ± 0.56 a	8.1 ± 0.38 a	9.1 ± 0.17 a	5.1 ± 0.50 b
	**DI**	22.1 ± 0.44 a	16.7 ± 0.34 a	13.3 ± 0.73 b	2.9 ± 0.07 b	5.9 ± 0.30 a	7.6 ± 0.44 b	6.6 ± 0.34 b	6.9 ± 0.73 b	6.1 ± 0.07 a
	**FI**	20.8 ± 0.52 b	13.7 ± 0.47 b	17.2 ± 1.26 a	8.0 ± 0.34 a	4.6 ± 0.34 b	6.8 ± 0.52 b	6.3 ± 0.47 b	5.8 ± 1.26 b	6.6 ± 0.34 a
**ECt**	**NI**	70.6 ± 0.76 a	79.7 ± 1.26 a	76.3 ± 0.15 a	83.8 ± 1.61 a	85.6 ± 3.63 a	80.3 ± 0.76 b	83.9 ± 1.26 b	81.7 ± 0.15 a	89.5 ± 1.61 a
	**DI**	69.8 ± 0.75 a	82.0 ± 1.35 a	72.9 ± 1.41 b	85.5 ± 0.68 a	83.5 ± 0.99 a	86.4 ± 0.75 a	87.9 ± 1.35 a	75.4 ± 1.41 b	86.1 ± 0.68 a
	**FI**	71.8 ± 1.68 a	75.9 ± 1.93 b	74.8 ±1.34 ab	78.7 ± 1.07 b	86.6 ± 2.11 a	80.3 ± 1.68 b	85.8 ± 1.93 ab	78.9 ± 1.34 ab	80.6 ± 1.07 b
**ECGt**	**NI**	8.7 ± 0.13 a	9.2 ± 0.20 a	12.8 ± 0.68 a	8.1 ± 0.26 c	7.9 ± 1.15 b	9.4 ± 0.13 b	8.0 ± 0.20 a	9.2 ± 0.68 b	5.4 ± 0.26 b
	**DI**	8.1 ± 0.13 b	1.3 ± 0.13 b	13.8 ± 0.63 a	11.6 ± 0.13 b	10.6 ± 0.32 a	6.0 ± 0.13 c	5.5 ± 0.13 c	17.8 ± 1.63 a	7.7 ± 0.13 b
	**FI**	7.5 ± 0.20 c	10.4 ± 0.31 a	7.9 ± 0.68 b	13.3 ± 0.43 a	8.8 ± 0.49 b	12.9 ± 0.20 a	7.9 ± 0.31 b	15.3 ± 0.68 a	12.8 ± 0.43 a
**Ce**	**NI**	3.5 ± 0.05 a	2.1 ± 0.12 c	2.6 ± 0.06 a	3.1 ± 0.09 b	2.6 ± 0.00 a	4.9 ± 0.05 a	2.8 ± 0.12 c	4.7 ± 0.06 c	4.9 ± 0.09 b
	**DI**	3.7 ± 0.06 a	5.4 ± 0.08 a	2.6 ± 0.05 a	10.3 ± 0.03 a	0.0 ± 0.00 b	4.1 ± 0.06 b	3.3 ± 0.08 b	6.0 ± 0.05 b	4.5 ± 0.03 b
	**FI**	3.6 ± 0.10 a	3.6 ± 0.08 b	2.0 ± 1.90 a	3.0 ± 0.03 b	0.0 ± 0.00 b	4.9 ± 0.10 a	4.8 ± 0.08 a	9.5 ± 1.90 a	11.4 ± 0.03 a
**ECe**	**NI**	93.8 ± 0.63 a	92.6 ± 1.46 a	92.9 ± 0.63 a	90.5 ± 2.10 a	89.4 ± 1.41 a	93.2 ± 0.63 a	95.2 ± 1.46 a	94.3 ± 0.63 a	93.5 ± 2.10 a
	**DI**	94.4 ± 2.27 a	81.3 ± 4.39 b	93.3 ± 2.24 a	77.0 ± 1.25 b	91.6 ± 1.46 a	94.1 ± 2.27 a	95.0 ± 4.39 a	92.1 ± 2.24 a	94.0 ± 1.25 a
	**FI**	93.5 ± 1.17 a	92.2 ± 1.10 a	93.1 ± 2.33 a	90.9 ± 0.95 a	87.7 ± 1.53 a	93.5 ± 1.17 a	93.5 ± 1.10 a	89.3 ± 2.33 a	87.2 ± 0.95 b
**ECGe**	**NI**	2.7 ± 0.02 a	5.3 ± 0.33 b	4.5 ± 0.01 a	6.5 ± 0.08 b	8.0 ± 0.08 b	1.9 ± 0.02 a	2.0 ± 0.03 a	0.9 ± 0.01 c	1.6 ± 0.08 a
	**DI**	1.9 ± 0.03 b	13.3 ± 0.06 a	4.1 ± 0.02 a	12.6 ± 0.02 a	8.4 ± 0.04 b	1.8 ± 0.03 a	1.7 ± 0.06 b	2.0 ± 0.02 a	1.5 ± 0.02 a
	**FI**	2.9 ± 0.12 a	4.2 ± 0.66 b	4.9 ± 0.32 a	6.1 ± 0.09 b	12.3 ± 0.06 a	1.7 ± 0.12 a	1.7 ± 0.07 b	1.2 ± 0.32 b	1.3 ± 0.09 b
**Polymers**										
**Ct**	**NI**	21.1 ± 0.10 a	18.6 ± 0.05 a	16.7 ± 0.09 b	16.7 ± 0.07 b	28.5 ± 0.13 b	5.3 ± 0.10 a	4.7 ± 0.05 a	4.2 ± 0.09 b	4.2 ± 0.07 c
	**DI**	20.0 ± 0.04 a	18.9 ± 0.06 a	20.1 ± 0.05 a	22.5 ± 0.04 a	48.7 ± 0.08 a	5.0 ± 0.04 a	4.7 ± 0.06 a	5.0 ± 0.05 a	5.6 ± 0.04 a
	**FI**	20.6 ± 0.16 a	13.5 ± 0.05 b	17.3 ± 0.04 b	19.9 ± 0.10 b	24.3 ± 0.02 b	5.1 ± 0.16 a	3.4 ± 0.05 b	4.3 ± 0.04 b	5.0 ± 0.10 b
**ECt**	**NI**	78.9 ± 0.10 a	81.4 ± 0.26 b	83.3 ± 0.17 a	83.3 ± 0.17 a	71.5 ± 0.11 a	94.7 ± 0.10 a	95.3 ± 0.26 a	95.8 ± 0.17 a	95.8 ± 0.17 a
	**DI**	80.0 ± 0.15 a	81.1 ± 0.43 b	79.9 ± 0.18 b	77.5 ± 0.20 b	51.3 ± 0.05 b	95.0 ± 0.15 a	95.3 ± 0.43 a	95.0 ± 0.18 a	94.4 ± 0.20 a
	**FI**	79.4 ± 0.18 a	86.5 ± 0.52 a	82.7 ± 0.18 a	80.1 ± 0.23 a	75.7 ± 0.07 a	94.9 ± 0.18 a	96.6 ± 0.52 a	95.7 ± 0.18 a	95.0 ± 0.23 a
**ECGt**	**NI**	nd	nd	nd	nd	nd	nd	nd	nd	nd
	**DI**	nd	nd	nd	nd	nd	nd	nd	nd	nd
	**FI**	nd	nd	nd	nd	nd	nd	nd	nd	nd
**Ce**	**NI**	1.0 ± 0.04 a	1.1 ± 0.05 a	1.0 ± 0.04 a	1.1 ± 0.09 a	1.1 ± 0.07 a	6.8 ± 0.04 b	14.1 ± 0.05 a	8.3 ± 0.04 b	1.3 ± 0.09
	**DI**	1.0 ± 0.03 a	1.1 ± 0.04 a	1.3 ± 0.15 a	1.2 ± 0.10 a	1.3 ± 0.03 a	5.6 ± 0.03 c	12.6 ± 0.04 b	8.9 ± 0.15 b	nd
	**FI**	1.0 ± 0.09 a	1.2 ± 0.05 a	1.0 ± 0.03 a	1.1 ± 0.04 a	1.0 ± 0.11 a	8.4 ± 0.09 a	14.8 ± 0.05 a	12.6 ± 0.03 a	nd
**ECe**	**NI**	56.7 ± 0.61 a	55.6 ± 0.60 a	57.7 ± 1.34 a	62.7 ± 1.73 a	64.5 ± 1.44 a	51.8 ± 0.61 b	42.6 ± 0.60 b	72.6 ± 1.34 a	81.5 ± 1.73 a
	**DI**	54.5 ± 0.86 a	53.8 ± 0.49 a	52.3 ± 1.05b	54.3 ± 1.20 b	59.2 ± 0.75 b	58.7 ± 0.86 a	51.5 ± 0.49 a	66.2 ± 1.05 b	73.6 ± 1.20 b
	**FI**	51.4 ± 1.42 b	50.5 ± 1.47 a	52.2 ± 1.31 b	53.9 ± 1.64 b	58.5 ± 0.53 b	48.4 ± 1.42 c	48.0 ± 1.46 a	60.7 ± 1.31 c	71.7 ± 1.64 b
**ECGe**	**NI**	0.2 ± 0.01 a	0.3 ± 0.03 a	0.4 ± 0.01 a	0.2 ± 0.03 b	0.3 ± 0.00 a	0.4 ± 0.01 b	0.4 ± 0.02 a	0.4 ± 0.01 a	0.3 ± 0.02 a
	**DI**	0.3 ± 0.01 a	0.2 ± 0.03 a	0.2 ± 0.03 b	0.2 ± 0.00 b	0.2 ± 0.02 a	0.6 ± 0.01 a	0.4 ± 0.02 a	0.3 ± 0.02 a	0.4 ± 0.01 a
	**FI**	0.3 ± 0.02 a	0.2 ± 0.02 a	0.1 ± 0.00 b	0.4 ± 0.03 a	0.3 ± 0.01 a	0.3 ± 0.01 b	0.4 ± 0.02 a	0.4 ± 0.02 a	0.2 ± 0.02 b
**EGCe**	**NI**	42.0 ± 0.60 b	42.9 ± 0.49 b	40.8 ± 1.21 b	36.0 ± 1.66 b	34.2 ± 1.49 b	41.0 ± 1.29 a	58.5 ± 1.03 a	18.7 ± 2.13 c	16.9 ± 0.44 b
	**DI**	44.3 ± 0.87 a	44.8 ± 0.41 b	46.2 ± 1.13 a	44.3 ± 1.06 a	39.3 ± 0.73 a	35.1 ± 0.51 b	35.5 ± 2.56 b	24.6 ± 0.89 b	26.0 ± 1.02 a
	**FI**	47.3 ± 1.20 a	48.0 ± 1.12 a	46.7 ± 1.27 a	44.6 ± 1.47 a	40.2 ± 0.42 a	42.9 ± 0.89 a	36.8 ± 1.61 b	36.3 ± 0.76 a	28.1 ± 2.33 a

^1^ Values with different letters within samplings and compounds are significantly different (Tukey’s test, *p* < 0.05).

**Table 3:** Seasonal evolution of the percentage of terminal and extension subunits in oligomeric and polymeric fractions of Syrah grape seeds from the three irrigation treatments (FI, 100% of ETc; DI, 50% of ETc and NI, non-irrigated) for 2011 and 2012. Terminal subunits: Ct, (+)-catechin; ECt, (−)-epicatechin; ECGt, epicatechin-3-*O*-gallate; extension subunits: %Ce, (+)-catechin; %ECe, (−)-epicatechin; %ECGe, epicatechin-3-*O*-gallate; nd, not detected.

		Day of 2011					Day of 2012			
		217	224	231	237	244	210	217	224	236
**Oligomers**										
**Ct**	**NI**	28.9 ±0.19 a ^1^	29.7 ± 0.10 b	30.2 ± 0.35 a	28.6 ± 0.14 b	29.6 ± 0.35 a	28.8 ± 0.15 a	25.5 ± 0.21 a	24.1 ± 0.21 a	25.5 ± 0.65 a
	**DI**	28.8 ± 0.45 a	31.4 ± 0.33 a	30.9 ± 0.12 a	32.3 ± 0.87 a	27.3 ± 0.48 b	28.0 ± 0.19 b	24.7 ± 0.10 b	24.9 ± 0.35 a	23.4 ± 0.14 b
	**FI**	27.9 ± 0.28 a	27.6 ± 0.23 c	27.2 ± 0.17 b	27.5 ± 0.30 b	26.9 ± 0.20 b	27.7 ± 0.16 b	21.4 ± 0.16 c	24.1 ± 0.29 a	22.9 ± 0.14 b
**ECt**	**NI**	42.5 ± 0.20 a	44.4 ± 0.22 b	47.3 ± 0.20 b	47.8 ± 0.11 b	51.5 ± 0.12 c	53.0 ± 0.11 a	58.6 ± 0.10 a	61.4 ± 0.63 a	61.6 ± 0.57 a
	**DI**	44.6 ± 0.93 a	44.2 ± 0.52 b	49.1 ± 0.39 a	46.9 ± 0.84 b	53.3 ± 0.95 b	52.4 ± 0.45 ab	59.0 ± 0.33 a	60.2 ± 0.12 a	62.5 ± 0.87 a
	**FI**	44.7 ± 0.08 a	48.1 ± 0.20 a	49.8 ± 0.04 a	53.6 ± 0.10 a	55.2 ± 0.18 a	51.4 ± 0.42 b	60.0 ± 0.50 a	62.5 ± 0.54 a	62.2 ± 0.58 a
**ECGt**	**NI**	28.6 ± 0.19 a	25.9 ± 0.16 a	22.5 ± 0.29 a	23.6 ± 0.14 a	18.9 ± 0.21 a	18.1 ± 0.23 b	15.9 ± 0.09 b	14.5 ± 0.18 a	12.9 ± 0.38 b
	**DI**	26.6 ± 0.45 a	24.4 ± 0.50 b	20.0 ± 0.54 b	20.8 ± 0.58 b	19.4 ± 0.14 a	19.6 ± 0.28 a	16.3 ± 0.23 b	14.8 ± 0.17 a	14.1 ± 0.30 a
	**FI**	27.4 ± 0.28 a	24.3 ± 0.26 b	23.0 ± 0.32 a	18.9 ± 0.24 c	18.0 ± 0.01 a	20.9 ± 0.27 a	18.6 ± 0.26 a	13.4 ± 0.32 a	14.9 ± 0.24 a
**Ce**	**NI**	9.3 ± 0.20 a	9.8 ± 0.14 a	9.3 ± 0.11 a	9.1 ± 0.07 a	9.5 ± 0.22 a	4.1 ± 0.10 a	4.7 ± 0.14 a	4.2 ± 0.18 a	4.2 ± 0.14 a
	**DI**	8.8 ± 0.93 b	9.6 ± 0.78 a	9.9 ± 0.60 a	9.4 ± 0.36 a	9.0 ± 0.25 b	3.8 ± 0.20 a	3.9 ± 0.22 b	4.2 ± 0.20 a	4.3 ± 0.11 a
	**FI**	9.1 ± 0.08 a	8.9 ± 0.09 b	8.9 ± 0.03 b	9.6 ± 0.07 a	8.9 ± 0.05 b	4.1 ± 0.18 a	3.8 ± 0.14 b	4.1 ± 0.11 a	4.2 ± 0.07 a
**ECe**	**NI**	88.7 ± 0.15 a	88.3 ± 0.21 a	88.8 ± 0.21 a	88.7 ± 0.65 a	88.3 ± 0.12 a	94.6 ± 0.40 a	94.0 ± 0.24 a	94.6 ± 0.71 a	94.4 ± 0.73 a
	**DI**	89.3 ± 0.11 a	88.7 ± 0.10 a	88.5 ± 0.63 a	88.6 ± 0.57 a	88.9 ± 0.16 a	94.9 ± 0.93 a	94.9 ± 0.52 a	94.5 ± 0.39 a	94.3 ± 0.84 a
	**FI**	89.0 ± 0.23 a	89.2 ± 0.09 a	89.0 ± 0.18 a	88.6 ± 0.38 a	89.0 ± 0.13 a	94.7 ± 0.97 a	94.9 ± 0.78 a	94.6 ± 0.60 a	94.5 ± 0.36 a
**ECGe**	**NI**	2.0 ± 0.10 a	1.8 ± 0.14 a	1.9 ± 0.18 a	2.2 ± 0.14 a	2.2 ± 0.05 a	1.3 ± 0.12 a	1.3 ± 0.11 a	1.3 ± 0.08 a	1.4 ± 0.09 a
	**DI**	1.9 ± 0.40 a	1.8 ± 0.24 a	1.6 ± 0.71 b	2.0 ± 0.73 a	2.1 ± 0.32 a	1.3 ± 0.08 a	1.2 ± 0.20 a	1.4 ± 0.04 a	1.4 ± 0.10 a
	**FI**	1.9 ± 0.12 a	1.9 ± 0.11 a	2.0 ± 0.08 a	1.8 ± 0.09 a	2.1 ± 0.07 a	1.3 ± 0.08 a	1.3 ± 0.09 a	1.3 ± 0.03 a	1.3 ± 0.07 a
**Polymers**										
**Ct**	**NI**	23.5 ± 0.12 a	31.6 ± 0.29 a	24.7 ± 0.33 c	32.8 ± 0.36 a	34.5 ± 0.10 a	28.3 ± 0.12 a	27.8 ± 0.29 a	25.8 ± 0.33 a	25.1 ± 0.36 a
	**DI**	23.8 ± 0.70 a	28.8 ± 0.19 b	29.9 ± 0.47 a	28.3 ± 1.42 a	32.7 ± 0.28 b	26.1 ± 0.70 b	25.8 ± 0.19 b	25.2 ± 0.47 a	24.6 ± 1.42 a
	**FI**	23.8 ± 0.63 a	24.8 ± 0.30 c	27.3 ± 0.17 b	33.4 ± 0.41 a	31.4 ± 0.07 b	27.0 ± 0.63 b	26.5 ± 0.30 b	26.7 ± 0.17 a	23.5 ± 0.41 a
**ECt**	**NI**	44.6 ± 0.53 a	41.5 ± 0.19 b	54.6 ± 0.43 a	40.9 ± 0.33 b	41.5 ± 0.13 b	52.7 ± 0.53 c	52.6 ± 0.19 c	56.8 ± 0.43 a	58.0 ± 0.33 b
	**DI**	44.2 ± 0.31 a	42.9 ± 0.18 b	43.0 ± 0.54 b	46.1 ± 0.75 a	42.7 ± 0.22 b	56.2 ± 0.31 b	56.7 ± 0.18 b	57.9 ± 0.54 a	61.1 ± 0.75 a
	**FI**	44.2 ± 0.32 a	47.4 ± 0.30 a	43.6 ± 0.21 b	44.0 ± 0.77 a	44.6 ± 0.23 a	58.9 ± 0.32 a	59.3 ± 0.30 a	57.1 ± 0.21 a	63.3 ± 0.77 a
**ECGt**	**NI**	31.9 ± 0.15 a	26.9 ± 0.15 a	20.7 ± 0.18 b	26.3 ± 0.09 a	24.0 ± 0.04 a	19.0 ± 0.15 a	19.5 ± 0.15 a	17.4 ± 0.18 a	16.9 ± 0.09 a
	**DI**	32.0 ± 0.37 a	28.2 ± 0.19 a	27.1 ± 0.41 a	25.6 ± 0.06 a	24.6 ± 0.17 a	17.7 ± 0.37 b	17.5 ± 0.19 b	16.9 ± 0.41 ab	14.3 ± 0.06 b
	**FI**	32.0 ± 0.43 a	27.7 ± 0.34 a	29.0 ± 0.14 a	22.6 ± 0.24 b	24.0 ± 0.11 a	14.1 ± 0.43 c	14.2 ± 0.34 c	16.2 ± 0.14 b	13.2 ± 0.24 b
**Ce**	**NI**	4.8 ± 0.17 a	4.9 ± 0.06 a	5.3 ± 0.13 a	5.1 ± 0.14 a	5.4 ± 0.14 a	6.7 ± 0.17 a	6.9 ± 0.06 a	6.7 ± 0.13 a	6.7 ± 0.14 a
	**DI**	4.6 ± 0.22 a	5.1 ± 0.06 a	4.5 ± 0.28 b	5.1 ± 0.16 a	4.8 ± 0.06 b	6.5 ± 0.22 a	6.7 ± 0.06 a	6.7 ± 0.28 a	6.8 ± 0.16 a
	**FI**	4.6 ± 0.08 a	4.1 ± 0.13 b	4.4 ± 0.08 b	5.0 ± 0.09 a	4.9 ± 0.05 b	6.4 ± 0.08 a	6.4 ± 0.13 a	6.5 ± 0.08 a	6.4 ± 0.09 a
**ECe**	**NI**	93.0 ± 0.43 a	93.0 ± 0.14 a	92.7 ± 0.24 a	92.6 ± 0.19 a	92.2 ± 0.22 a	92.2 ± 0.43 a	91.9 ± 0.14 a	92.1 ± 0.24 a	92.1 ± 0.19 a
	**DI**	93.1 ± 0.49 a	92.9 ± 0.15 a	93.9 ± 1.03 a	92.5 ± 0.71 a	92.7 ± 0.25 a	92.4 ± 0.49 a	92.2 ± 0.15 a	92.2 ± 1.03 a	92.0 ± 0.71 a
	**FI**	93.1 ± 0.51 a	93.7 ± 0.16 a	93.4 ± 0.29 a	92.7 ± 0.56 a	92.6 ± 0.31 a	92.6 ± 0.51 a	92.5 ± 0.16 a	92.4 ± 0.29 a	92.5 ± 0.56 a
**ECGe**	**NI**	2.2 ± 0.08 a	2.1 ± 0.07 a	2.0 ± 0.04 b	2.4 ± 0.06 a	2.4 ± 0.02 a	1.1 ± 0.08 a	1.2 ± 0.07 a	1.1 ± 0.04 a	1.2 ± 0.06 a
	**DI**	2.3 ± 0.11 a	2.0 ± 0.10 a	1.6 ± 0.04 c	2.4 ± 0.10 a	2.4 ± 0.08 a	1.1 ± 0.11 a	1.1 ± 0.10 a	1.1 ± 0.04 a	1.2 ± 0.10 a
	**FI**	2.3 ± 0.13 a	2.1 ± 0.05 a	2.3 ± 0.10 a	2.3 ± 0.07 a	2.5 ± 0.07 a	1.0 ± 0.13 a	1.0 ± 0.05 a	1.1 ± 0.10 a	1.2 ± 0.07 a

^1^ Values with different letters within samplings and compounds are significantly different (Tukey’s test, *p* < 0.05).

## Supplementary Materials

Supplementary materials are available online.
